# The correlates of appearance focused self-concept: personality traits, self-concept, sociocultural, and early life experience factors

**DOI:** 10.1186/s40337-024-01065-1

**Published:** 2024-08-02

**Authors:** Catherine Sarginson, Juliana Nicoletta, Thalia Charlebois, Sarah Enouy, Nassim Tabri

**Affiliations:** Department of Psychology, Carleton University, 1125 Colonel By Drive, Ottawa, ON K1S 5B6 Canada

**Keywords:** Personality traits, Perfectionism, Sociocultural, Self-concept, Appearance focused self-concept

## Abstract

**Background:**

Theory and research indicate that an appearance focused self-concept (i.e*.*, placing overriding importance on physical appearance for self-definition and self-worth) plays a role in the etiology and maintenance of disordered eating and eating disorders. Although the consequences of an appearance focused self-concept are palpable, less is known about its correlates. Accordingly, we examined a range of factors that may characterize appearance focused people, including personality traits (perfectionism, impulsivity, sensation-seeking, hopelessness, and anxiety sensitivity), self-concept (global self-esteem and self-concept clarity), sociocultural (thin-ideal, muscular-ideal, general attractiveness internalizations, and perceived pressure to be thin), and early life experiences (adverse childhood experiences, attachment styles) factors.

**Methods:**

Female undergraduate university students (*N* = 568; *M*_age_ = 19.58, *SD*_age_ = 4.24) completed a questionnaire battery that included the Beliefs About Appearance Scale, Depressive Experiences Questionnaire–Self-Criticism-6 Scale, Frost-Multidimensional Perfectionism Scale, the Revised Almost Perfect Scale, Substance Use Risk Profile Scale, Self-Concept Clarity Scale, Rosenberg Self-Esteem Scale, Sociocultural Attitudes Towards Appearance Questionnaire-4 Scale, Adverse Childhood Experiences Questionnaire, Experiences in Close Relationships Scale Short Form, and the Dietary Restraint subscale of the Eating Disorders Examination Questionnaire.

**Results:**

Multiple regression analyses were conducted for each set of factors separately and together. For personality traits, perfectionism, impulsivity, and anxiety sensitivity were uniquely associated with appearance focused self-concept. For self-concept, global self-esteem and self-concept clarity were uniquely associated with appearance focused self-concept. For sociocultural, general attractiveness internalization, thin-ideal internalization, and perceived pressure to be thin were uniquely associated with appearance focused self-concept. For early life experiences, attachment anxiety and avoidance were uniquely associated with appearance focused self-concept. In the combined analysis, the various factors explained 54% of the variance in appearance focused self-concept. Impulsivity, global self-esteem, general attractiveness internalization, and perceived pressure to be thin were uniquely associated with appearance focused self-concept.

**Conclusions:**

Results for global self-esteem were consistent with prior research. Findings for evaluative concerns perfectionism were inconsistent with prior research. We discuss future research directions to examine the link between evaluative concerns perfectionism and appearance focused self-concept. We also discuss how sociocultural factors (general attractiveness internalization and perceived pressure to be thin) and impulsivity may help cultivate an appearance focused self-concept, advancing knowledge on the characteristics of appearance-focused people.

## Abstract (highlights)

It has been proposed that people who place more (relative to less) importance on appearance in their self-concept are more likely to engage in disordered eating and to have eating disorders, namely anorexia nervosa and bulimia nervosa. Yet, little is known about the characteristics of people who place more (relative to less) importance on appearance in their self-concept. Accordingly, we examined how the importance people give to appearance in their self-concept may be linked to various aspects of their personality, self-perception, societal influences, and early life experiences. Questionnaires were given to 568 female university students—a group that is known to be vulnerable to disordered eating and eating disorders. We found that some factors were connected to how much importance is placed on appearance. In particular, feeling pressure to be thin, wanting to be attractive, thinking poorly of oneself, and being impulsive were all linked to giving appearance more importance in the self-concept. These findings help us learn more about the people who view appearance as central in their lives. Understanding these connections can help us support people living with disordered eating and eating disorders.

## Introduction

Most people derive self-definition and self-worth from their perceived performance in multiple life domains, including interpersonal relationships with friends and family, vocational achievements, financial success, and appearance to name a few [1, 2]. The different life domains typically vary in perceived importance and so a person’s self-concept is judged against performance in life domains that are perceived to be more important to them [2, 3]. Critically, people who overvalue the importance of a single life domain as a core aspect of their self-concept may experience mental and physical health problems [4–6]. People who have an appearance focused self-concept (i.e*.*, they overvalue the importance of physical appearance for self-definition and self-worth) are more likely to engage in disordered eating and to have eating disorders (e.g., [7–9]). In the Transdiagnostic Cognitive-Behavioural Theory of Eating Disorders [10], an appearance focused self-concept is positioned as the core psychopathology that maintains disordered eating among people with anorexia nervosa (AN) and bulimia nervosa (BN). Likewise, theory and research indicate that an appearance focused self-concept may play a role in the maintenance of binge eating disorder (BED; [10, 11]). Furthermore, in the fifth edition of the Diagnostic and Statistical Manual of Mental Disorders [12, 13], an appearance focused self-concept is a diagnostic criterion for AN and BN, and a severity specifier for BED.

Although the consequences of an appearance focused self-concept are palpable, little is known about its correlates. To our knowledge, only two studies exist that examined the correlates of appearance focused self-concept among adolescent girls [14, 15]. Of note, different correlates were examined in these studies. Wade and Lowes [14] examined perceived conflict between parents, comments from family members about appearance, perfectionism, and low global self-esteem, and found that they each had a moderate correlation with appearance focused self-concept. In analyses that controlled shared variance, only perfectionism, global self-esteem, and comments about appearance had unique correlations with appearance focused self-concept. Wilksch and Wade [15] examined temperament, including sensitivity to punishment and sensitivity to reward, and sociocultural factors (i.e., thin-ideal internalization, and perceived pressure to be thin), which had small-to-moderate positive correlations with appearance focused self-concept. Given the dearth of research, we explored a wide array of correlates of appearance focused self-concept, including personality traits (perfectionism, impulsivity, sensation seeking, hopelessness, and anxiety sensitivity), self-concept (global self-esteem and self-concept clarity), sociocultural (thin-ideal, muscular-ideal, general attractiveness internalizations and perceived pressure to be thin), and early life experience (adverse childhood experiences and attachment styles) factors.

### Personality traits

In the Transdiagnostic Cognitive-Behavioral Theory of Eating Disorders [10], perfectionism is a risk factor for an appearance focused self-concept. Perfectionism is a multidimensional construct involving self-imposed high personal standards, evaluative concerns or self-criticism, and the overvaluation of striving [16, 17]. Prior research has shown that different dimensions of perfectionism have moderately positive cross-sectional correlations with appearance focused self-concept in student, community, and clinical samples [14, 17–22]. Also, findings from longitudinal research indicate that personal standards and evaluative concerns were prospectively correlated with appearance focused self-concept over time among adolescent girls [23, 24]. Together, these findings support the role of perfectionism as a risk factor for an appearance focused self-concept.

Other personality traits that have been linked with eating disorders may also play a role in the development of an appearance focused self-concept. Wilksch and Wade [15] found an association between the temperament of sensitivity to punishment and appearance focused self-concept among adolescent girls. People with greater sensitivity to punishment tend to avoid stimuli, environments, or experiences that elicit negative affect, including feelings of fear and anxiety (e.g., [23]). According to Gray [25, 26], sensitivity to punishment underpins the personality domain of neuroticism, which includes broad dispositions to experience negative cognitions and affect. Hopelessness and anxiety sensitivity are two lower order dimensions of neuroticism [27–29] that may be correlated with appearance focused self-concept. Existing evidence indicates that hopelessness is negatively associated with body regard [30] and positively associated with negative views about physical appearance [31] among adolescents. Likewise, anxiety sensitivity was shown to be positively associated with the drive for thinness among university students and in a sample of university students seeking outpatient treatment for mental health problems [32]. It is possible that people high in fear of bodily sensations and interoceptive experiences, which characterize anxiety sensitivity, may also place more importance on their appearance as a source of self-definition and self-worth. Although no prior research has examined the relation between hopelessness and anxiety sensitivity on the one hand and appearance focused self-concept on the other hand, the available evidence suggests that they are likely correlated.

Furthermore, the temperament of sensitivity to reward has been shown to be positively associated with an appearance focused self-concept among adolescent girls [15]. People with greater sensitivity to reward tend to pursue pleasant and rewarding stimuli, environments, and experiences that elicit feelings of elation and satisfaction. The sensitivity to reward temperament underpins impulsivity because it pertains to a propensity toward immediate gratification [33]. Of note, impulsivity (e.g., [34]) and sensation seeking (e.g., [33, 35, 36])—a facet of impulsivity that refers to a tendency toward novel stimuli and experiences—have both been associated with eating disorders. To our knowledge, however, no research has examined whether impulsivity and sensation seeking are both associated with appearance focused self-concept. With that said, findings from research on a focused self-concept in other life domains have shown that greater trait impulsivity is linked with having a financially focused self-concept among people with gambling problems [37]. Perhaps people who score higher (relative to lower) on impulsivity and/or sensation seeking may be more focused on a specific area of life for self-definition and self-worth.

### Self-concept

Two aspects of the self-concept that may be correlated with appearance focused self-concept are global self-esteem (GSE) [2] and self-concept clarity (SCC) [38]. GSE refers to the extent a person has a general positive attitude towards themselves. In the Transdiagnostic Cognitive-Behavioral Theory of Eating Disorders [10], low GSE is positioned as a risk factor for developing an appearance focused self-concept. Cross-sectional research has found moderately negative associations between GSE and appearance focused self-concept in samples of high school [14] and university [19] students.

SCC refers to a person’s beliefs that the content of their self-concept is clearly defined, internally consistent, and temporally stable [38]. Vartanian [39] proposed that people with lower (relative to higher) SCC may be more likely to turn to appearance to inform their self-definition and self-worth. In support of his proposition, Vartanian [39] showed a moderately negative association between SCC and basing self-worth on appearance in a sample of university students.

To our knowledge, no other research exists that specifically examined the link between SCC and GSE on the one hand and appearance focused self-concept on the other hand.

### Sociocultural

Although the role of sociocultural factors in the etiology of eating disorders is relatively well-established (for a review, see [40]), much less is known about whether sociocultural factors are correlated with appearance focused self-concept. Sociocultural factors include the internalization of societal attractiveness ideals (i.e., they buy into the notion that attaining the attractiveness ideal will bring them happiness and life success) and perceived pressure to attain the attractiveness ideal from family, friends, peers, and dating partners, as well as from society via mass media (for a review, see [40]. In their cognitive model of anorexia nervosa, Basten and Touyz [41] positioned thin-ideal internalization as a risk factor for the development of an appearance focused self-concept. Likewise, it has been shown in prior research that a moderately positive association exists between thin-ideal internalization and appearance focused self-concept in samples of adolescent girls [15] and university students [42]. Yet, no research has examined the internalization of other appearance ideals, such as the athletic-ideal, or the internalization of general attractiveness (see [40]). Similarly, no research has examined the link between perceived pressure to attain the attractiveness ideal in relation to appearance focused self-concept.

Based on the sociocultural model of the self-concept [43], sociocultural factors (internalization and perceived pressure) may be risk factors for an appearance focused self-concept. Indeed, the content of the self-concept includes ideas about who one is (the actual self), who one wants to become (the ideal self), and evaluative judgments about oneself that inform self-worth. Furthermore, the content of people’s self-concept is shaped, in part, by input from interpersonal relationships and from prevailing societal norms of their cultural milieu. In other words, how people understand and evaluate themselves and who they aspire to be are influenced, in part, by their beliefs about the expectations of important people they interact with (e.g., parents, peers, and dating partners) have for them and by culture-specific societal standards of success and happiness in life (e.g., being thin is the appearance standard of attractiveness that brings happiness and life success for women in Western cultures). Accordingly, internalization of attractiveness and perceived sociocultural pressure to attain the attractiveness ideal may each contribute to cultivating an appearance focused self-concept.

### Early life experiences and attachment styles

It has been proposed that adverse childhood experiences (ACEs; e.g., emotional and physical abuse and neglect, and family dysfunction) are linked to the development of an appearance focused self-concept [44]. ACEs have been linked to eating disorders as well as various psychiatric conditions in adulthood [45]. Also, various aspects of ACEs have been moderately linked to eating, shape, and weight concerns in community and clinical samples [46, 47]. To our knowledge, however, no prior research has examined the link between ACEs and appearance focused self-concept specifically.

Another aspect of early life experience that may underpin the development of an appearance focused self-concept in adulthood is insecure attachment styles. Attachment styles are different ways of interacting and behaving in interpersonal relationships that stem from early caregiver-child interactions. Insecure attachment styles stem from inconsistencies in caregivers’ responses to a child’s needs, which may manifest in adulthood as either an anxious attachment style or an avoidant attachment style [48]. An anxious attachment style refers to a disposition to desire close relationships and fear abandonment. An avoidant attachment style refers to a disposition to avoid close relationships and to be distant and aloof in relationships. Amianto and colleagues [49] proposed that insecure attachment styles may play a role in the etiology of eating disorders because they interfere with the development of the self. Likewise, Monteleone and colleagues [50] proposed that people with an insecure attachment style may develop an appearance focused self-concept because insecure attachment styles promote the use of the body as a source of self-definition. Critically, although there is some research that has linked insecure attachment styles with weight concerns [51], no prior research has specifically examined the link between insecure attachment styles and appearance focused self-concept specifically.

## Overview of the current research

Herein, we examined the observed and unique associations between appearance focused self-concept on the one hand and an array of personality traits, self-concept, sociocultural, and early life experiences on the other hand. We examined these associations in a large sample of university women—a population in which the prevalence of disordered and eating and eating disorders is elevated relative to the general population [52].

## Method

### Participants and procedure

A total of 738 female undergraduate students participated in the current research. The data of 170 participants were excluded from the analyses due to missing data. Thus, the data of 568 participants were examined. They were recruited through the Psychology Department’s recruitment pool to complete questionnaires and were compensated with course credit. All participants provided informed consent and the research was approved by Carleton University Research Ethics Board-B.

### Measures

Participants reported their height and weight in inches and pounds, respectively, which we used to compute their body mass index (BMI). They also completed the following measures. Note that unless otherwise indicated, higher scores on each measure reflect more of the measured construct.

#### Dietary restraint

The Dietary Restraint subscale of the Eating Disorders Examination-Questionnaire (EDE-Q) [53] was used to characterize participants’ tendency towards disordered eating. Participants responded to five items (e.g., “On how many days of the past 28 days have you gone for long periods of time (8 waking hours or more) without eating anything at all in order to influence your shape or weight?”). Participants responded to each item using a 7-point response scale with endpoints *no days* (0) and *every day* (7). An average score was calculated (α = 0.89).

#### Appearance focused self-concept

The Beliefs About Appearance Scale (BAAS; [42]) was used to measure the extent to which people have an appearance focused self-concept. Herein, we used four items from the original 20 items from the BAAS that have been used in prior research [6, 54]. The items were: “How I feel about myself is largely based on my appearance”; “My moods are influenced by how I look”; “People will think less of me if I don’t look my best”; and “The opportunities that are available to me depend upon how I look”. Participants responded to each item using a 5-point response scale with endpoints *not at all* (0) and *extremely* (4). An average score was calculated (α = 0.82).

#### Perfectionism

Perfectionism was measured using 24 items drawn from the Depressive Experience Questionnaire–Self-Criticism (DEQ-SC6; [55]), Frost Multidimensional Perfectionism Scale (Frost-MPS; [56]) and the Revised Almost Perfect Scale (APS; [57]). Participants indicated the degree to which they agreed or disagreed with each statement using a 7-point Likert scale with endpoints *strongly disagree* (1) and *strongly agree* (7). This combination of measures has been used in prior research to assess personal standards and self-critical dimensions of perfectionism [21, 58].

Results of an exploratory factor analysis indicated that two separate factors underlie the 28 items. Nine items loaded on the first factor we labelled personal standards (e.g., “It is important to me that I be thoroughly competent in everything I do”). Seven items loaded onto a second factor we labelled self-criticism perfectionism (e.g*.*, “Doing my best never seems to be enough”). The remaining items did not load strongly on either factor and so were excluded. Average scores were computed for personal standards perfectionism (α = 0.92) and evaluative concerns perfectionism (α = 0.90).

#### Impulsivity, sensation seeking, hopelessness, and anxiety sensitivity

The 23-item Substance Use Risk Profile Scale (SURPS; [29]) was used to assess impulsivity (6 items; e.g*.*, “Generally, I am an impulsive person”), sensation seeking (5 items; e.g., “I would like to skydive”), hopelessness (7 items; e.g., “I feel that I’m a failure”), and anxiety sensitivity (5 items; e.g*.*, “I get scared when I’m too nervous”). Participants responded to each item using a scale with end points *strongly disagree* (1) and *strongly agree* (7). Average scores were computed for impulsivity (α = 0.76), sensation seeking (α = 0.73), hopelessness (α = 0.90), and anxiety sensitivity (α = 0.69).

#### Self-concept clarity (SCC)

Participants completed the 12-item SCC Scale [38] (e.g*.*, “My beliefs about myself often conflict with one another”). They responded to each item using a 7-point Likert scale with endpoints *strongly disagree* (1) and *strongly agree* (7). An average score was calculated (α = 0.88).

#### Global self-esteem

The Rosenberg Self-Esteem Scale [59] was used to measure global self-esteem. Participants indicated the extent to which they agreed with 10 items (e.g*.*, “I take a positive attitude towards myself”) using a 7-point Likert scale with endpoints *strongly disagree* (1) and *strongly agree* (7). An average score was calculated (α = 0.87). Note that we extended the response scale from 4-points to 7-points.

#### Sociocultural factors

The 15-item Sociocultural Attitudes Towards Appearance Questionnaire-4 (SATAQ-4; [60]) was used to measure thin-ideal (e.g., “I think a lot about looking thin”), general attractiveness (e.g., “It is important to me to be attractive”), and muscular-ideal (e.g., “It is important for me to look muscular”) internalizations, as well as perceived pressure to be thin from peers (e.g.*,* “I feel pressure from my peers to improve my appearance”), family (e.g*.*, “Family members encouraged me to decrease my level of body fat”), and significant others (e.g*.*, “I feel pressure from significant others to improve my appearance”). Participants indicated the degree to which they agree with each statement using a 7-point Likert scale with endpoints *strongly disagree* (1) and *strongly agree* (7). Average scores were computed for thin-ideal (α = 0.83), attractiveness-ideal (α = 0.88), muscular-ideal (α = 0.92) internalizations and perceived pressure to be thin (α = 0.93).

#### Adverse childhood experiences (ACEs)

A 28-item scale [61, 62] was used to measure ACEs that occurred before the age of 18. The subscales assessed emotional (e.g., “While you were growing up, how often did a parent, stepparent, or adult living in your home swear at you, insult you, or put you down?”) and physical (e.g., “While you were growing up, how often did a parent, stepparent, or adult living in your home push, grab, shove, slap, or throw something at you?”) abuse, as well as neglect (e.g*.*, “While you were growing up you didn’t have enough to eat?”). There were also subscales for sexual abuse (e.g., “During the first 18 years of your life, did an adult or older relative, family friend, or stranger ever touch or fondle you in a sexual way?”), and household dysfunction (e.g., “While you were growing up, was anybody in your household depressed or mentally ill?”). In the 28-item measure, some items had a binary response option (*yes* = 1 and *no* = 0) and other items had a 5-point response scale with endpoints *never* (1) and *very often* (5). In line with Dong et al. [63], responses that used the 5-point scale were dichotomized with “often” or “very often” being coded as *yes* (1) and scores less than four being coded as *no* (0). A total score was calculated across all items that ranged from zero (i.e., no ACEs) to 10 (i.e., experienced all types of ACEs) before the age of 18 (α = 0.84).

#### Attachment styles

The 12-item Experiences in Close Relationships Scale Short Form (ECR-S; [64]) was used to measure attachment anxiety (e.g., “I need a lot of reassurance that I am loved by my partner”) and attachment avoidance (e.g*.*, “I don’t feel comfortable opening up to romantic partners”). Participants indicated their level of agreement with anxiety and avoidance subscale items using a 7-point Likert scale with endpoints *strongly disagree* (1) and *strongly agree* (7). Total scores were computed for attachment anxiety (α = 0.89) and attachment avoidance (α = 0.87).

### Statistical analyses

Five analyses were conducted using SPSS version 28. First, descriptive analyses were conducted to characterize the sample in terms of age and BMI, as well as risk for disordered eating using the dietary restraint subscale of the EDE-Q. Second, a regression analysis was conducted to examine the relationship between the personality traits (perfectionism, hopelessness, anxiety sensitivity, impulsivity, and sensation seeking) and appearance focused self-concept. Third, a regression analysis was conducted to examine the self-concept factors (global self-esteem and self-concept clarity) and appearance focused self-concept. Fourth, a regression analysis was conducted to examine the relation between sociocultural factors (thin-ideal internalization, muscularity internalization, general attractiveness internalization, and perceive pressure to be thin) and appearance focused self-concept. Fifth, a regression analysis was conducted to examine the relation between early life experiences (attachment anxiety, attachment avoidance, and ACEs), and appearance focused self-concept. Sixth, a regression analysis was conducted to examine the relation between personality traits, self-concept, sociocultural, and early life experiences simultaneously on the one hand and appearance focused self-concept on the other hand.

For each of the five regression analyses, we verified the regression assumptions in terms of multivariate outliers, multicollinearity, and heteroscedasticity. These assumption checks are located on the Open Science Framework (OSF). Briefly, there was evidence of heteroscedasticity only for the regression analysis examining the link between self-concept factors and appearance focused self-concept. As such, we used robust Huber-White *SE*s for this regression analysis. For all other regression analyses, normal theory *SE*s were used.

### Transparency statement

In the interest of openness and transparency, the analyzed data and statistical output files are available on the Open Science Framework: https://osf.io/8zwsv/.

## Results

### Sample characteristics

Participants were between the ages of 17 and 68 (*M* = 19.58, *SD* = 4.24) years old. Note that 95.4% of the sample were between 17 and 25 years old. Eight participants did not report their height or weight, or both. Among those who did, BMI ranged from 10.18 to 54.81 (*M* = 23.54, *SD* = 4.99). Three participants did not respond to one of the dietary restraint items and so their mean score was based on their available responses. The mean score for dietary restraint in the sample was 1.83 (*SD* = 1.70), which was slightly higher than the normative dietary restraint score for female undergraduate students in the US [65]. Using a cut-off score of ≥ 4.0 for clinical significance [65], 13.7% of the sample scored in the clinically significant range.

### Personality traits

Descriptive statistics and observed correlations are in Table 1. The personality traits were all positively correlated with appearance focused self-concept. The largest correlation was between self-critical perfectionism and appearance focused self-concept, which was moderate in size. The regression model explained 30% of the variance in appearance focused self-concept, adjusted *R*^2^ = 0.30, *F*(6, 567) = 40.68, *p* < 0.001. Self-critical and personal standard perfectionism as well as anxiety sensitivity and impulsivity were uniquely associated with appearance focused self-concept (see Table 2). Hopelessness and sensation seeking were not uniquely associated with appearance focused self-concept.

**Table 1:**
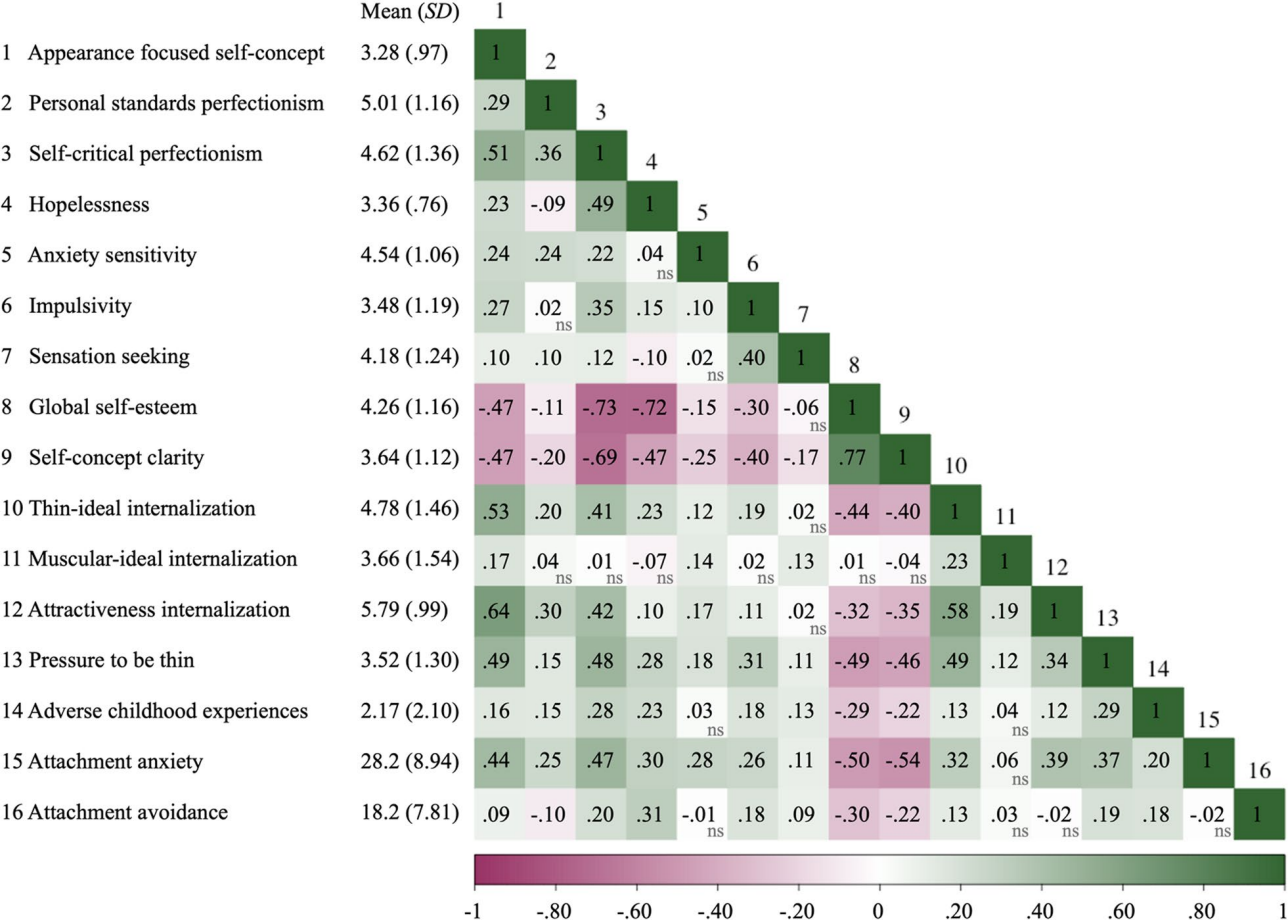
Correlations between all measured variables

All associations are statistically significant (*p* < 0.05) unless otherwise indicated with the label ns

**Table 2:** Results for predicting appearance focused self-concept from personality traits

Independent variable	*B*(*SE*)	95% CI_*B*_	β	*P* value
Perfectionism—personal standards	0.129(0.036)	[0.059, 0.199]	0.149	< 0.001
Perfectionism—self-criticism	0.243(0.033)	[0.179, 0.307]	0.363	< 0.001
Hopelessness	0.042(0.055)	[− 0.066, 0.150]	0.033	0.445
Anxiety sensitivity	0.103(0.034)	[0.036, 0.169]	0.112	0.002
Impulsivity	0.105(0.033)	[0.040, 0.171]	0.129	0.002
Sensation seeking	< 0.001(0.031)	[− 0.036, 0.064]	< 0.001	0.994

### Self-concept

Global self-esteem and self-concept clarity were both negatively associated with appearance focused self-concept (see Table 1). The magnitude of the observed associations was moderate in size. The regression model explained 25% of the variance in appearance focused self-concept, adjusted *R*^2^ = 0.25, *F*(2, 567) = 94.22, *p* < 0.001. Global self-esteem and self-concept clarity were each uniquely associated with appearance focused self-concept (see Table 3).

**Table 3:** Results for predicting appearance focused self-concept from self-concept factors

Independent variable	*B*(*SE*)	95% CI_*B*_	β	*P* value
Global self-esteem	− 0.220(.049)	[− 0.315, − 0.125]	− 0.262	< 0.001
Self-concept clarity	− 0.233(.054)	[− 0.331, − 0.135]	− 0.269	< 0.001

### Sociocultural

Thin-ideal internalization, muscular ideal internalization, general attractiveness internalization, and perceived pressure to be thin were positively associated with appearance focused self-concept (see Table 1). The magnitude of the associations was moderate in size, except for the association between muscular-ideal internalization and appearance focused self-concept, which was small-to-moderate in size. The regression model explained about 50% of the variance in appearance focused self-concept, adjusted *R*^2^ = 0.49, *F*(4, 567) = 139.24, *p* < 0.001. Thin-ideal internalization, general attractiveness internalization, and perceived pressure to be thin were each uniquely associated with appearance focused self-concept (see Table 4). Muscular-ideal internalization was not uniquely associated with appearance focused self-concept.

**Table 4:** Results for predicting appearance focused self-concept sociocultural factors

Independent variable	*B*(*SE*)	95% CI_*B*_	β	*P* value
Thin-ideal internalization	0.080(0.027)	[0.028, 0.133]	0.120	0.003
Muscular-ideal internalization	0.015(0.020)	[− 0.023, 0.054]	0.024	0.436
General attractiveness internalization	0.468(0.037)	[0.396, 0.540]	0.473	< 0.001
Perceived pressure to be thin	0.198(0.026)	[0.147, 0.249]	0.263	< 0.001

### Early life experience factors

Attachment anxiety, attachment avoidance, and ACEs were positively associated with appearance focused self-concept (see Table 1). The magnitude of the association for attachment anxiety was moderate, whereas the magnitude of the association for attachment avoidance and ACEs was small. The regression model explained 20% of the variance in appearance focused self-concept, adjusted *R*^2^ = 0.20, *F*(3, 567) = 49.24 *p* < 0.001. Attachment anxiety and attachment avoidance were each uniquely associated with appearance focused self-concept (see Table 5). ACEs were not uniquely associated with appearance focused self-concept.

**Table 5:** Results for predicting appearance focused self-concept from early life experiences

Independent variable	*B*(*SE*)	95% CI_*B*_	β	*P* value
Adverse childhood experiences	0.028(0.018)	[− 0.007, 0.064]	0.061	0.118
Attachment anxiety	0.047(0.004)	[0.039, 0.055]	0.430	< 0.001
Attachment avoidance	0.011(0.005)	[0.001, 0.020]	0.085	0.026

### Combined analysis

The regression model explained 54% of the variance in appearance focused self-concept, adjusted *R*^2^ = 0.54, *F*(15, 567) = 45.12, *p* < 0.001. General attractiveness internalization had a moderate unique association with appearance focused self-concept, whereas perceived pressure to be thin, impulsivity, and global self-esteem had small unique associations with appearance focused self-concept (see Table 6). No other unique associations were statistically significant.

**Table 6:** Results for predicting appearance focused self-concept from personality traits, self-concept, sociocultural, early life experiences

Independent variable	*B*(*SE*)	95% CI_*B*_	β	*P* value
Perfectionism—personal standards	0.056(0.029)	[− 0.001, 0.112]	0.067	0.053
Perfectionism—self-criticism	0.035(0.035)	[− 0.035, − 0.104]	0.048	0.328
Hopelessness	− 0.014(0.057)	[− 0.127, 0.099]	− 0.011	0.809
Anxiety sensitivity	0.055(0.028)	[− 0.001, 0.111]	0.060	0.053
Impulsivity	0.060(0.029)	[0.004, 0.116]	0.074	0.035
Sensation seeking	0.014(0.026)	[− 0.036, 0.064]	0.018	0.579
Global self-esteem	− 0.121(0.055)	[− 0.228, − 0.014]	− 0.144	0.027
Self-concept clarity	0.002(0.044)	[− 0.084, 0.089]	0.003	0.958
Thin-ideal internalization	0.051(0.026)	[− 0.001, 0.103]	0.076	0.055
Muscular-ideal internalization	0.026(0.019)	[− 0.012, 0.064]	0.041	0.176
General attractiveness internalization	0.405(0.038)	[0.331, 0.479]	0.410	< 0.001
Perceived pressure to be thin	0.116(0.028)	[0.062, 0.170]	0.154	< 0.001
Adverse childhood experiences	− 0.016(0.014)	[− 0.045, 0.012]	− 0.035	0.259
Attachment anxiety	0.007(0.004)	[− 0.001, 0.015]	0.063	0.089
Attachment avoidance	0.001(0.004)	[− 0.007, 0.009]	0.007	0.837

## Discussion

The findings of the current research indicate that appearance focused self-concept is associated with a range of personality traits, self-concept, sociocultural, and early life experiences among university women—a group that is vulnerable to eating disorders. Although over 50% of the variance in appearance focused self-concept was explained, not all factors had a unique association with appearance focused self-concept. Results are discussed below in terms of their implications.

### Implications

Of the personality traits assessed, evaluative concerns perfectionism had a large observed association with appearance focused self-concept. Evaluative concerns perfectionism was also uniquely associated with appearance focused self-concept in analyses that controlled for shared variance with other personality traits. These findings are consistent with theory and prior research. However, we found that evaluative concerns perfectionism was not uniquely associated with appearance focused self-concept when controlling for shared variance with self-concept, sociocultural and early life experience factors. The absence of a unique association may reflect the presence of a more complex link between evaluative concerns perfectionism and appearance focused self-concept. It is possible that the link between perfectionism and appearance focused self-concept may be contingent on the presence of appearance concerns. For example, findings from experimental research supports a causal relation between perfectionism and appearance focused self-concept among women high, but not low, in body dissatisfaction [23]. It is also possible that there exists a multiplicative effect of perfectionism and appearance focused self-concept on eating disorder risk [16]. For instance, in a longitudinal study, undergraduate women had the highest levels of disordered eating when they had high levels of both perfectionism and appearance focused self-concept compared to women who had differential levels or low levels of perfectionism and appearance focused self-concept [17]. Accordingly, a potentially important future direction for research is to examine whether perfectionism is an antecedent of appearance focused self-concept among people with appearance concerns, a moderator of the link between appearance focused self-concept and disordered eating, or both.

We also observed that impulsivity had a moderate observed association with appearance focused self-concept. Impulsivity was also uniquely associated with appearance focused self-concept in analyses that controlled for shared variance with other personality traits. The unique association between impulsivity and appearance focused self-concept was also present but attenuated when controlling for shared variance with self-concept, sociocultural, and early life experience factors. These findings are consistent with prior research showing a positive association between the temperament of sensitivity to reward and appearance focused self-concept among adolescent girls [15]. The findings for impulsivity are also consistent with theory and correlational research examining a focused self-concept in other domains, namely a financially focused self-concept [37, 66]. Perhaps people who are more (relative to less) impulsive are more focused on a specific area of life. Thus, a potential future direction for research is to examine the mechanisms by which impulsivity may focus the self-concept on a single area of life.

Of the remaining personality traits, anxiety sensitivity was uniquely associated with appearance focused self-concept in analyses that controlled for shared variance with other personality traits. In analyses that also controlled for shared variance with self-concept, sociocultural, and early life experience factors, the statistical significance of the unique association for anxiety sensitivity was marginal and thus should be interpreted with caution. That said, the findings for anxiety sensitivity extend prior research linking anxiety sensitivity with other aspects of eating disorder psychopathology, including body dissatisfaction and interoceptive awareness among undergraduate students [32]. Of the remaining personality traits, sensation seeking and hopelessness had observed, but not unique, associations with appearance focused self-concept.

As for self-concept factors, SCC and GSE had moderate observed associations with appearance focused self-concept. SCC and GSE also had moderate unique associations when controlling for their shared variance. However, only GSE had a unique association with appearance focused self-concept when controlling for SCC, as well as the personality traits, sociocultural, and early life experience factors, which were small-to-moderate in size. The findings for GSE are consistent with the Transdiagnostic Cognitive-Behavioural Theory of Eating Disorders, wherein GSE is positioned as a risk factor for the development of an appearance focused self-concept. The findings for GSE also replicate and extend prior cross-sectional research among high school and university students, which found moderately negative observed associations between GSE and appearance focused self-concept [14, 67]. To our knowledge, however, no longitudinal research exists and so it remains unresolved whether low GSE is an antecedent, consequence, or correlate of appearance focused self-concept, or a combination of these factors.

In terms of sociocultural factors, general attractiveness internalization and thin-ideal internalization, as well as perceived pressure to be thin had moderately positive observed associations with appearance focused self-concept, whereas muscular-ideal internalization had a small observed association. These findings are consistent with prior research that found small-to-moderate associations between thin-ideal internalization and perceived pressure to be thin on the one hand and appearance focused self-concept on the other hand among adolescent girls [15]. In analyses that controlled for the shared variance between the sociocultural factors, only muscular-ideal internalization was no longer associated with appearance focused self-concept. However, muscular-ideal and thin-ideal internalization were not uniquely associated with appearance focused self-concept when controlling for shared variance with other sociocultural factors, as well as personality traits, self-concept, and early life experience factors. Note that the level of statistical significance for the unique association between thin-ideal internalization and appearance focused self-concept was marginal and so should be interpreted with caution.

In contrast, general attractiveness internalization and perceived pressure to be thin had unique associations with appearance focused self-concept. The magnitude of the unique association for general attractiveness internalization was moderate-to-large. A potential reason for the robust link between general attractiveness internalization and an appearance focused self-concept is that people who score higher (relative to lower) on appearance focused self-concept have an attentional bias for words that describe attractiveness—not words that describe stigmatized appearance (e.g., fat) and general descriptors of appearance (e.g., body; [68]). Another potential reason is that appearance focused people tend to attribute benefits to attractiveness, such as feeling happier and more self-confident, as well as having greater ease with finding a romantic partner and with vocational achievement [69]. Therefore, it would behoove researchers to examine the temporal direction of the link between general attractiveness internalization and appearance focused self-concept using a longitudinal research design.

Similarly, the unique association for perceived pressure to be thin (from family, friends, dating partners, and mass media) was small-to-moderate. This finding is novel, and we recommend further examination of the link between perceived pressure to be thin and appearance focused self-concept. Because sociocultural attractiveness ideals are ubiquitous in everyday life, such as through interactions with peers, as well as through content and images in print and online media, it is possible that how much an individual perceives pressure to be thin may vary moment-to-moment, which may influence appearance focused self-concept on a daily basis. Accordingly, a potentially important future research direction would be to examine the causal role of perceived pressure to be thin in the development of an appearance focused self-concept using experience sampling methodology.

ACEs and insecure attachment styles had observed associations with appearance focused self-concept. The observed association for ACEs was small. One reason for the small association may be related to the type of ACEs being measured. Although the ACEs measure that we used focused on events involving general abuse, a recent review of the literature suggests that family-related non-abuse experiences may be important for understanding eating disorders (e.g*.,* family disharmony, adverse parenting style; [70]). Indeed, one study found that perceived conflict between parents and receiving comments about appearance from family members were both positively associated with appearance focused self-concept among adolescent girls [14]. Thus, it may be important in future research to distinguish between general abuse and family-related non-abuse ACEs in relation to appearance focused self-concept.

In analyses that controlled for shared variance between the early life experience factors, only attachment anxiety and attachment avoidance had unique associations with appearance focused self-concept. However, in analyses that controlled for shared variance with personality traits, self-concept, and sociocultural factors, none of the early life experience factors had a unique association with appearance focused self-concept. The absence of unique associations for ACEs and insecure attachment styles may be because they are *distal* transdiagnostic etiological factors for various psychiatric conditions (e.g., [71]). According to Nolen-Hoeksema and Watkins [72], an important goal of theory and future research on distal transdiagnostic factors is to identify the moderating factors and mediating mechanisms that cultivate unique forms of psychopathology. Relatedly, Crocker and Park [73] proposed that people focus on life domains for self-definition and self-worth that they believe offers them protection and safety from dangers perceived in childhood. As such, for instance, experiencing more traumatic and difficult appearance-related events in childhood and adolescents, such as weight teasing and bullying by family members and peers, may be a potential proximal mechanism by which experiencing abusive and non-abusive ACEs cultivate an appearance focused self-concept. Future research can examine these possibilities.

### Limitations

A limitation of the current research is the cross-sectional design. As such, causal inferences cannot be made from the results. Future research can use experimental and longitudinal research designs to help determine the nature of the relationship between appearance focused self-concept and the other measured variables. Another possible limitation is the nature of the sample, which were female university students. Thus, the external validity of the results to people of other genders, sexes, and ages is unknown. It is also unclear whether the results would generalize to people who do not have access to university. Accordingly, future research should aim to replicate and extend the findings of the current research using more demographically diverse samples.

### Conclusions

The current research advances knowledge on the characteristics of people with an appearance focused self-concept. Of note, appearance focused self-concept was correlated with a range of personality, self-concept, sociocultural, and early life experiences. We also observed that two sociocultural factors—general attractiveness internalization and perceived pressure to be thin—GSE, and impulsivity were uniquely associated with appearance focused self-concept. These results suggest that sociocultural factors, GSE, and impulsivity may be important for understanding the etiology and maintenance of an appearance focused self-concept.

## Data Availability

The analyzed data and statistical output files are available via the Open Science Framework: https://osf.io/8zwsv/.
